# Stimuli-Responsive Liposomes of 5-Fluorouracil: Progressive Steps for Safe and Effective Treatment of Colorectal Cancer

**DOI:** 10.3390/pharmaceutics16070966

**Published:** 2024-07-22

**Authors:** Hamad Alrbyawi

**Affiliations:** Department of Pharmaceutics and Pharmaceutical Industries, College of Pharmacy, Taibah University, Madinah 41477, Saudi Arabia; hrbyawi@taibahu.edu.sa

**Keywords:** 5-fluorouracil, colorectal cancer, targeted drug delivery systems, stimuli-responsive liposomes

## Abstract

5-Fluorouracil (5-FU) has become one of the most widely employed antimetabolite chemotherapeutic agents in recent decades to treat various types of cancer. It is considered the standard first-line treatment for patients with metastatic colorectal cancer. Unfortunately, traditional chemotherapy with 5-FU presents many limitations, such as a short half-life, a low bioavailability, and a high cytotoxicity, affecting both tumor tissue and healthy tissue. In order to overcome the drawbacks of 5-FU and enhance its therapeutic effectiveness against colorectal cancer, many studies have focused on designing new delivery systems to successfully deliver 5-FU to tumor sites. Liposomes have gained attention as a well-accepted nanocarrier for several chemotherapeutic agents. These amphipathic spherical vesicles consist of one or more phospholipid bilayers, showing promise for the drug delivery of both hydrophobic and hydrophilic components in addition to distinctive properties, such as biodegradability, biocompatibility, a low toxicity, and non-immunogenicity. Recent progress in liposomes has mainly focused on chemical and structural modifications to specifically target and activate therapeutic actions against cancer within the proximity of tumors. This review provides a comprehensive overview of both internal-stimuli-responsive liposomes, such as those activated by enzymes or pH, and external-stimuli-responsive liposomes, such as those activated by the application of a magnetic field, light, or temperature variations, for the site-specific delivery of 5-FU in colorectal cancer therapy, along with the future perspectives of these smart-delivery liposomes in colorectal cancer. In addition, this review critically highlights recent innovations in the literature on various types of stimuli-responsive liposomal formulations designed to be applied either exogenously or endogenously and that have great potential in delivering 5-FU to colorectal cancer sites.

## 1. Introduction

Colorectal cancer (CRC) is the third most common cancer globally after lung and breast cancer and the second most fatal cancer after lung cancer. In 2020, there were more than 1.9 million cases of CRC, and more than 930,000 deaths due to CRC were estimated to have occurred, which accounts for about 10% of the global cancer incidence and 9.4% of cancer fatalities [1]. By 2030, it is estimated that the global burden of CRC will rise to over 2.2 million new cases and 1.1 million deaths [2]. Based on the projection of human development, population growth, and aging, the global number of new CRC cases is anticipated to reach 3.2 million in 2040. The prospect for CRC therapy is usually good. The 5-year survival rate of patients with early CRC is approximately 90% [3]. However, the symptoms of CRC often appear in the advanced stages, which explains the differences in the survival rate. The early detection of CRC is a crucial determinant in reducing mortality and improving both the prognosis and the quality of future life. In addition to the early detection of CRC, heterogeneity in the survival rates can be explained by tumor characteristics, the host response, and the treatment approach (radiotherapy, immunotherapy, chemotherapy, targeted therapy, or surgery).

Advances in our understanding of CRC pathophysiology have resulted in increased treatment options. While surgery remains the cornerstone treatment for early-stage CRC, chemotherapy is the primary treatment option for metastatic disease when the tumor is not fully resectable at presentation [4]. The available chemotherapeutic agents for metastatic CRC are 5-fluorouracil (5-FU), oxaliplatin, and irinotecan. 5-FU has become one of the most widely employed antimetabolite chemotherapeutic agents for patients with metastatic CRC. The overall survival rate for 5-FU-based chemotherapy has been firmly established. The survival rate in elderly patients (>70 years) and young patients (<70 years) was found to be equal (10.8 months and 11.3 months, respectively) when patients received a 5-FU-containing treatment [5]. 5-FU is a water-soluble drug; hence, it can be administered as an intravenous infusion or bolus. However, the overall response rate to 5-FU in advanced colorectal cancer is limited to 10–15%, owing to its low bioavailability due to rapid metabolism (10–20 min) and acquired resistance [6]. Moreover, 5-FU results in severe adverse events, such as myelosuppression, stomatitis, and pulmonary embolism [7]. Thus, various drug delivery systems (DDSs) have been developed for 5-FU to minimize its side effects and achieve a better therapeutic effect in the treatment of CRC.

Currently, great attention is being paid by researchers to nanotechnology-based drug delivery systems for the treatment of CRC, such as nanotubes, lipid-based drug delivery systems, inorganic nanoparticles, and nanorods [8,9]. Nanotechnology-based drug delivery systems are able to overcome the limitations of conventional medication therapy, as they have the ability to increase the concentration of the drug in the site of action and reduce the risk of adverse events. Stimuli-responsive drug delivery platforms have attained the greatest attention because these drug platforms respond to specific triggers to release their load at the desired site; hence, they can enhance the drug’s efficacy and minimize its adverse effects. Stimuli-responsive drug delivery platforms have attained more attention due to their potential applications in the treatment of CRC. Drug delivery through stimuli-responsive platforms involves triggering drug release at the target site in response to chemical or physical stimuli [10]. The insensitivity of stimuli-responsive drug delivery platforms toward normal cells prevents the release of chemotherapeutic drugs and, therefore, alleviates the off-target events associated with chemotherapy. Several stimuli-responsive drug delivery platforms have been developed to target 5-FU toward CRC in order to achieve selective improvement of tumor necrosis. This review focuses on the stimuli-responsive drug delivery platforms that have been fabricated to deliver 5-FU as chemotherapy to treat CRC. It provides insight into physical-stimuli-responsive drug delivery platforms and chemical-stimuli-responsive drug delivery platforms for the triggered release of 5-FU through active and passive targeting in CRC sites. In addition, this review critically highlights the recent innovations in and the literature on various types of stimuli-responsive liposomal formulations, which are designed to be applied either exogenously or endogenously and have great potential in delivering 5-FU to colorectal cancer sites.

## 2. 5-Fluorouracil: Mechanism of Action and Resistance Pathways in Colorectal Cancer

5-Fluorouracil (5-FU) is a fluorinated derivative of pyrimidine that is widely used for the treatment of cancer, particularly for CRC. The fluoropyrimidine 5-FU is converted inside cells into fluorodeoxyuridine monophosphate, fluorodeoxyuridine triphosphate, and fluorouridine triphosphate, which are the active metabolites of 5-FU [11]. 5-FU exerts its antitumor effects through the incorporation of its metabolites into RNA and DNA and through the inhibition of thymidylate synthase (TS) [12]. TS is involved in the regulation of protein synthesis and apoptotic processes, as it is an essential enzyme for catalyzing the biosynthesis of thymidylate [13]. On the other hand, over 80% of the 5-FU administered is catabolized by dihydropyrimidine dehydrogenase (DPD) to dihydrofluorouracil, which is an inactive metabolite [14].

The mechanisms of 5-FU resistance in CRC are numerous and complex, and they can occur simultaneously when cells are exposed to the drug. Some mechanisms are evolutionarily conserved, such as drug efflux, whereas others are specific to CRC, such as the tumor microenvironment. Resistance to 5-FU explains the low 5-year survival rate and the higher doses needed to treat CRC. Since over 80% of the 5-FU administered is catabolized by DPD in the liver and colorectal cells, a high expression level of DPD is consequently associated with 5-FU resistance [15].

## 3. Liposomes for Cancer Drug Delivery

**Figure 1:**
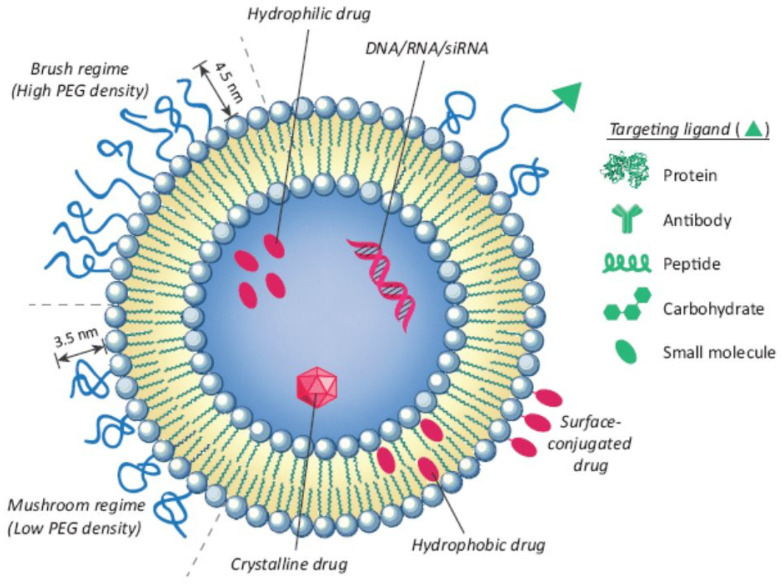
Structural features of liposomes as nanocarriers with an entrapped molecule. Functionalization of the liposome surface can be performed by binding with ligands, such as antibodies and proteins, to enhance receptor-mediated endocytosis [23]. (Reprinted with permission from Elsevier. A higher resolution/color version of this figure is available in the electronic copy of the article).

Liposomes are spherical vesicles composed of one or more layers of phospholipid bilayers, with each layer separated by an aqueous space. Their unique structure enables them to carry both water-soluble (hydrophilic) and fat-soluble (hydrophobic) therapeutic agents, either within aqueous compartments or embedded in the lipid bilayer itself [16]. Liposomes can vary considerably in size, typically falling within the range of 25 nanometers to 2.5 μm [17]. Liposomes are categorized according to two main characteristics: the number of bilayers they contain and their overall size. Single-layered liposomes are called unilamellar and have only one phospholipid bilayer, while multilamellar liposomes possess multiple layers, much like an onion’s structure. As drug vehicles, liposomes exhibit outstanding properties, such as an ability to extend the half-life of drug molecules (PEGylated liposomes), the protection of encapsulated substances from physiological degradation [18], the control of the release of drug molecules, and excellent safety and biocompatibility [19]. Liposomes can accumulate in tumor tissues passively due to their enhanced permeability and retention effect. Tumors often have a leaky vasculature and poor lymphatic drainage, allowing the relatively large liposome particles to enter and remain in the tumor tissue while normal tissues do not permit the same level of accumulation [20]. Furthermore, liposomes can be modified with specific ligands (Figure 1), such as antibodies, peptides, and small molecules, that can bind to target receptors expressed on the surface of cancer cells [21]. Liposomes can be designed to respond to specific internal stimuli in the target environment (e.g., pH, enzymes) or external stimuli (e.g., light, magnetic field) for on-site drug release [22].

## 4. Stimuli-Responsive Drug Delivery Platforms as a Potential Strategy to Improve 5-FU-Based Chemotherapy

One of the most important purposes of pharmaceutical research is to discover and design novel formulations and drug delivery systems to maximize therapeutic efficacy and minimize side effects. Novel nanocarriers and drug delivery platforms have the potential to precisely target cancer, enhance bioavailability, and reduce systemic toxicity. However, the early release of the drug before reaching the target tumor is one of the major challenges in novel drug delivery systems developed for cancer treatment [24]. Stimuli-responsive drug delivery platforms have revolutionized chemotherapy-based treatments, as they release their content only when they are triggered with a stimulus; as a result, they prevent the release of cytotoxic drugs into normal cells [25].

Stimuli-responsive drug delivery systems are able to control drug release in response to specific stimuli, either endogenous (changes in enzyme concentration, pH, or redox gradients) or exogenous (variations in the magnetic field, temperature, ultrasound intensity, or electric pulses) [26]. Functionalized modules and materials, including modern biomaterials such as peptides, nano-drug delivery systems, and polymers, exhibit an endogenous or exogenous responsive behavior due to their biomimetic nature. The application of these endogenous or exogenous stimuli is responsible for the disruption or alteration of the structure of specifically designed drug delivery systems, which results in the abrupt release of the enclosed drug [27].

The tumor microenvironment (TME) of CRC has extracellular factors, such as its enzymatic activity, acidity, and hypoxia, that make the design of novel 5-FU stimuli-responsive drug delivery platforms a promising strategy for targeting colorectal cancer [28]. Enormous changes in the physicochemical properties occur at the tissue and cellular levels as the tumor progresses. Insufficient nutrient transport modifies the tumor’s energy metabolism. Extracellular metabolite lactic acid reduces the pH in the tumor tissue and the rapid proliferation of tumor cells induces hypoxia in the tumor microenvironment [29]. Exogenous-based stimuli for 5-FU stimuli-responsive drug delivery platforms are a promising approach for targeting colorectal cancer since they possess the advantages of simple handling and a highly controllable treatment time and location [30]. When multiple stimuli-responsive strategies are integrated into a system, they offer a good platform for the co-delivery of 5-FU with other agents and the reversal of multidrug resistance in CRC.

## 5. Internal-Stimuli-Responsive Liposomes for 5-FU Delivery

### 5.1. pH-Responsive Liposomes

The variation in the pH values in the CRC region can provide a suitable physiological stimulus for pH-responsive liposomal drug delivery platforms in order to deliver 5-FU to the target area. The key principle of pH-responsive drug delivery systems is to release 5-FU when the pH trigger point is achieved. An acidic extracellular pH is a major feature of tumor tissues. The “Warburg effect” theory states that tumors tend to produce lactic acid through the anaerobic glycolytic pathway rather than through oxidative phosphorylation, even in the presence of sufficient oxygen [31]. The extracellular pHs of solid tumors such as those in CRC range between 6.0 and 7.0 [32]. Tumor cells also upregulate certain proteins, such as the Na^+^/H^+^ exchanger isoform 1 (NHE1), which are responsible for the acidification of the extracellular tumor microenvironment [32]. 

One way to achieve this kind of release system is by incorporating ionizable chemical groups, such as carboxylic acid and amines, with nanoparticles. Moreover, pH-sensitive polymers are commonly employed for the development of stimuli-responsive drug delivery systems. These polymers, which are polyelectrolytes, consist of ionizable groups within their backbones, side groups, or end groups. Udofot et al. [33] reported pH-sensitive liposomes for 5-FU based on cholesterylhemisuccinate (CHEMS), cholesterol (CH), tween 20, and 1,2-distearoyl-sn-glycero-3-phosphoethanolamine-N-[amino(polyethyleneglycol)-2000] (DSPE-PEG2000) in a molar ratio of 60:20:10:10, respectively. CHEMS has been found to destabilize the liposomal membrane under acidic pH conditions. This destabilization is believed to promote the accumulation of 5-FU at the tumor site. The liposomal nanoparticles exhibited a stronger anticancer effect compared to 5-FU against HT-29 and HCT-116 colon cancer cells. Enhanced antitumor effects observed with liposomal 5-FU formulations could be substantially attributed to the higher delivery rate of 5-FU, as well as the rapid breakdown of the liposomes in acidic environments, leading to a more effective cancer treatment. Approximately 35% of the 5-FU was released from the liposomal formulation within the initial 100 min at a pH level of 3. 

Banerjee et al. [34] presented the development of 5-FU liposomes anchored with pH-responsive polymers, poly(styrene-co-maleic acid) (SMA), to provide a more effective method for making vesicles sensitive to pH. SMA, an artificial copolymer, is recognized for its ability to transform into a neutral, globular shape in acidic environments as a result of alterations to its ionization levels. 5-FU-loaded pH-sensitive liposomes, composed of 1,2-distearoyl-sn-glycero-3-phosphocholine (DSPC) and SMA in a 20:1 molar ratio, demonstrated a higher cytotoxicity than loaded liposomes without SAM. The median inhibition concentration (IC_50_) values in HT-29 cells were found to be 6.54 and 8.16 M for 5-FU SMA-anchored liposomes and neat liposomes, respectively. The enhanced toxic effect of 5-FU could likely be attributed to the effective release of the medication from the liposomes, prompted by the alteration in the pH within the endosomes. When the calcein fluorescence de-quenching test was conducted to evaluate the co-polymer’s effect on the liposome’s membrane permeability and structural integrity, nearly 60% of the calcein was released within 10 min during incubation at a temperature of 37 °C and a pH of 5.0. 

**Figure 2:**
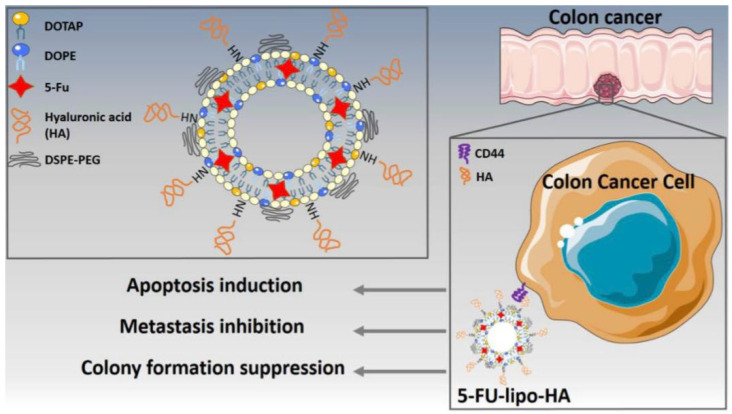
Hyaluronic acid surface-modified pH-sensitive liposomes encapsulating 5-FU were formulated for targeting the CD44 receptor in colorectal cancer cells [35]. (Reprinted with permission from John Wiley & Sons. A higher resolution/color version of this figure is available in the electronic copy of the article).

Mansoori et al. [35] developed hyaluronic acid (HA)-modified liposomes encapsulating 5-fluorouracil and tested them against a CD44-expressing colorectal cell line (HT29) (Figure 2). Liposomal nanoparticles were prepared by incorporating 1,2-dioleoyl-3-trimethylammonium-propane (DOTAP), 1,2-dioleoyl-sn-glycerol-3phosphoethanolamine (DOPE), 1,2-distearoyl-sn-glycero-3-phosphoethanolamine-N-[biotinyl(polyethylene glycol)-2000] (DSPE-PEG(2000)), and hyaluronic acid (HA) in a 1:1:1:0.5 molar ratio, respectively. Approximately 40% of the 5-FU was released from the liposomes over a period of 10 h in an acidic medium (pH 5.5, which is similar to endosomal pH), compared to 19% within the same 10 h duration at a physiological pH (7.4). DOPE plays a vital role in the structure of pH-sensitive liposomes. The exposure of DOPE-containing liposomes to acidic conditions leads to their destabilization. This is driven by the low hydration levels of DOPE’s polar head group, which transitions into a hexagonal inverted phase. This change results in the creation of non-lamellar formations that initiate the destabilization of the liposomal bilayers in acidic environments [36].

Shahidi et al. [37] prepared 5-FU-loaded liposomes by combining hydrogenated soybean phosphatidylcholine (HSPC), cholesterol, and 1,2-dipalmitoyl-sn-glycerol-3-phosphoglycerol (DPPG) (7:2:1 M ratio). Furthermore, the prepared liposomes were fabricated to be multilayered through a sequential assembly of cationic chitosan (CS), miR and siRNA, CS, and sodium hyaluronate (HA) layers onto the surface of the negatively charged cores. A study on the release of 5-fluorouracil was conducted using sterile phosphate-buffered saline (PBS) at two different pH levels: a pH of 5.4, to mimic the acidic environment of lysosomes, and a pH of 7.4, to represent the normal physiological conditions of the human body. Minimal release of 5-FU was observed in the PBS buffer at a pH of 7.4, with less than 50% dispersion over 80 h. Conversely, at a pH of 4.5, approximately 80% of the 5-FU was released over a comparable duration. The cell viability, assessed using an MTT assay on SW480 colon cancer cells, indicated that the liposomes had a remarkable cytotoxicity relative to free 5-FU. The in vivo antitumor efficacy of the liposomal formulation was examined on the tumor xenograft model using the CT26 colorectal carcinoma cell line. The liposomal formulation most efficiently reduced the tumor volume compared to free 5-FU (*p* < 0.01), and it did not show remarkable changes in body weight, indicating the good tolerance of this liposomal formulation. Chitosan, a cationic copolymer containing D-glucosamine and N-acetyl glucosamine, possesses superior features, including biocompatibility, biodegradability, and pH sensitivity. Chitosan is a pH-sensitive polymer due to the variation in its charge density at a pH below its pKa (around 6.5). At a lower pH, chitosan’s amine groups are protonated, which might cause liposomal membrane destabilization [38].

Several organic functional groups, lipids, chemical bonds, and inorganic compounds, which exhibit significantly different physicochemical properties in response to pH variations and which have been used to fabricate pH-responsive liposomes to deliver various chemotherapeutic agents for the treatment of different cancer types, might exhibit promising approaches for 5-FU delivery to CRC sites (Table 1). pH-sensitive liposomes can be modified with targeting ligands to specifically direct the liposomes toward cervical cancer (HeLa) cells [39]. Folate-receptor-targeted pH-sensitive liposomes, composed of HSPC/CHOL/mPEG2000-DSPE/folate–PEG3350-CHEMS in a molar ratio of 55:40:4:1, demonstrated the efficient and stable encapsulation of imatinib. These liposomes also showed enhanced drug release at a lower pH value (5.5), as folate–PEG3350-CHEMS decreases the rigidity and stability of the liposome bilayer at an acidic pH. Folate-receptor-targeted pH-sensitive liposomes showed an increased internalization efficiency and cytotoxicity, as folate–PEG3350-CHEMS could effectively target HeLa cells via the folate receptor. The formation of liposomes using oleic acid (OA) is a common method for changing the pH sensitivity of a liposome formulation. A liposomal formulation composed of phosphatidylethanolamine (PE)/cholesterol/oleic acid (OA)/docetaxel (DTX) in a ratio of 3:2:3:1 *w*/*w* achieved a 1.3-fold higher cumulative DTX release rate at a pH of 5.0 than at a pH of 7.4 [40]. As a negatively charged lipid, OA modified the intermolecular interactions of PE and destabilized the lipid bilayer, causing it to revert to the unstable hexagonal phase.

The chemical modification of liposomal formulations with a degradable hydrazine-linked PEG polymer was introduced as a promising approach to deliver various chemotherapeutic agents under acidic conditions. Such a conjugate is susceptible to hydrolysis in an acidic medium because it contains carbon–nitrogen double bonds. Chen et al. [41] investigated paclitaxel (PTX) pH-sensitive liposomes containing mPEG2000-Hz-CHEMS as the primary conjugate. The liposomal formulation composed of S100PC/Chol/mPEG2000-Hz-CHEMS/PTX (90:10:3:3) was stable at a physiological pH (t_1/2_: 40.9 h), while it was easily degraded at a pH of 5.5 (t_1/2_: 6.7 h). The modified mPEG2000-Hz-CHEMS liposomes did not increase the liver and spleen accumulation compared to the same formulation without hydrazone linkages. Amine functional groups have also been used as sites for protonation and deprotonation to induce pH-dependent changes in the permeability within liposomes. Hao et al. [42] introduced pH-sensitive bola-type triblock copolymers made of poly(2-(diisopropylamino) ethylmethacrylate) (PDPA) and mPEG segments. DOPC-based PDPA liposomes demonstrated the most significant doxorubicin (DOX) release profiles, with over 80% of the DOX released at a pH of 6.0 compared to only 20% at a pH of 7.4 after 48 h. Under acidic conditions, PDPA copolymers become more hydrophilic due to the protonation of di-isopropyl tertiary amines, which triggers DOX release.

The structural modification of liposomes through highly stable polymer-caged nanobins (PCNs) is another approach to trigger the release of chemotherapeutic agents under acidic conditions. Lee et al. [43] prepared PCNs via the insertion of cholesterol-modified poly(acrylic acid) (Chol-PAA) into liposomes loaded with DOX. Under acidic conditions (pH = 5–6), the PCN-DOX formulations transferred DOX more efficiently into the cytoplasm of KB human epithelial nasopharyngeal carcinoma cells and OvCa432 epithelial ovarian carcinoma cells due to the conformational collapse upon the protonation of the free acrylate groups. Another structural modification of liposomes was made through acid-cleavable mPEG-vinyl ether-1,2-dioleylglycerol lipids (mPEG-VE-DOG) in conjugation with DOPE [44]. The acid-catalyzed hydrolysis of the vinyl ether linkages produced a faster release rate of calcein (>95% at a pH of 3.5, and 60% at a pH of 4.5). Another liposomal formulation was designed using phenyl-substituted-vinyl-ether-(PIVE)–PEG-DOG conjugates and DOPE [45]. The liposomal formulation was stable at a physiological pH; however, it exhibited pH-induced dePEGylation and content release at an acidic pH (pH of 3.5 or 4.5). 

In another approach, pH-responsive liposomes were designed through the incorporation of aspartic acid, which initiated phase transitions upon ionization [46]. Furthermore, pH-sensitive liposomes modified with a sulfadimethoxine-based copolymer and a 3-methylglutarylated hyperbranched poly(glycidol) polymer that initiated phase transitions upon ionization under acidic conditions have been reported [47,48]. pH-responsive liposomes encapsulated with bicarbonate ions (NH_4_HCO_3_) were prepared. Encapsulating liposomes with bicarbonate ions resulted in CO_2_ effervescence upon acidification, which triggered drug release by creating pores in the liposome membrane [49].

### 5.2. Enzyme-Responsive Liposomes

The levels of several enzymes are elevated in various types of cancer, including colon cancer. This biochemical abnormality can be exploited as the trigger to design liposomes that undergo structural alterations, leading to the release of the encapsulated therapeutic payloads. In CRC, proteolytic enzymes, such as cysteine proteinases, serine proteinases, and metalloproteinases, play a major role in colorectal CRC invasion and metastasis [50]. In addition, lipase, glycosidase, and phosphatase enzymes have been found to be dysregulated in CRC. Designing liposomes with a linker that will cleave off in the presence of overexpressed enzymes is one approach for obtaining enzyme-sensitive liposomes. In a different approach, liposomes are stabilized with a polymer layer that can be broken down by a particular enzyme present at the tumor site. Liposomal prodrugs have gained significant attention for their capability of releasing drugs in response to enzymes. This method involves liposomes with drug compounds that are chemically bonded to the bilayer’s lipids. Once the linker is cleaved by an enzyme at the specific site, the active medication is liberated from the liposomes. Enzyme-responsive liposomes have the advantage of being stable in the physiological environment until activated at the tumor site by a specific enzyme [51]. 

Jin et al. [52] prepared a nanoassembly that contained 1-O-octadecyl-2-(5-fluorouracil)-N-acetyl-3-zidovudine-phosphorylglycerol (OFZG), which is a prodrug of 5-FU, to achieve the release of the drug at the targeted site depending on the sPLA_2_ enzyme. The bilayer nanoassemblies were composed of OFZG/CH/tween 80 (2:1:0.1 molar ratio). The in vitro experiments revealed that sPLA_2_ degraded OFZG and the formulation exhibited higher anticancer activity than the parent drug 5-FU in COLO205 and HCT-116 colon cancer cells. The IC_50_ values for the nanoassemblies were 2.01 and 17.4 M in COLO205 and HCT-116 cells, respectively. The IC_50_ values for 5-FU were 6.50 and 113 M, respectively. Following their introduction into mice with tumors, the nanostructures demonstrated a level of anticancer effectiveness similar to that of 5-FU, despite having just one-tenth of the molar concentration of 5-FU.

In addition to sPLA2, several enzymes in the CRC area are overexpressed, which can be exploited as endogenous triggers to attain 5-FU site-specific delivery. Matrix metalloprotease (MMP)-sensitive liposomes constitute one such promising target that is being explored for designing drug-targeting systems [53]. Within the MMP family, MMP2 and MMP9 are the most frequently targeted for drug delivery, as they play a role in the metastasis of various tumors, including colorectal, breast, lung, prostate, and ovarian cancers. Xu et al. [54] reported docetaxel-loaded liposomes, with reductions and MMP9 enzyme-sensitive properties, to enhance the docetaxel release and antitumor activity against 4T1 breast cancer cells. The MMP9-sensitive copolymer, methoxy polyethylene glycol-peptide-vitamin E succinate, and methoxy polyethylene glycol-s-s-vitamin E succinate were used to develop the liposomal formulation. Docetaxel-loaded liposomal delivery systems demonstrated a significantly higher antitumor efficacy and antimetastatic effects against 4T1 breast cancer cells compared to free drugs.

Another intracellular enzyme, elastase, has been linked to tumor progression and development due to its specificity for uncharged amino acid side chains, primarily alanine or valine. The covalent attachment of DOPE to an elastase substrate (N-acetyl-ala-ala) produced a cleavable peptide–lipid (N-Ac-AA-DOPE) and led to the formation of bilayer liposomes. The cleavage of the peptide–lipid (N-Ac-AA-DOPE) by elastase caused the destabilization and fusion of the liposomes as DOPE reverted to its original inverted cone structure [55]. Intracellular proteases such as cathepsin that are found in the lysosomal acidic environment offer a means for targeted delivery within cells. Cathepsin B-responsive liposomes composed of DOTAP/DPPC and coated with the polymeric lipid PEG-GLFG-K(C16)2 were designed to deliver doxorubicin [56]. The liposomal formulation showed cathepsin responsiveness and enhanced its anticancer effect on Hep G2 cells, inhibiting cell viability by 60% compared to 30% of free DOX at 5 µM. Researchers have also investigated numerous enzymatically triggered liposomes, including phospholipase C [57] and glucose oxidase [58].

## 6. External-Stimuli-Responsive Drug Delivery System for 5-FU

### 6.1. Thermosensitive Liposomes

Thermosensitive liposomes are a specialized type of liposome designed to release their encapsulated contents in response to an increase in temperature. These liposomes are engineered to be stable at body temperature (around 37 °C) but become permeable or disintegrate to release their contents when exposed to slightly higher temperatures (usually between 39 °C and 42 °C). This temperature-responsive behavior is achieved by carefully selecting and combining lipids (e.g., DPPC) that have phase transition temperatures within this range [59]. The mechanism of action for thermosensitive liposomes involves the lipid bilayer transitioning from a gel-like state to a more fluid state as the temperature rises above the lipid’s phase transition temperature (T_c_). In the gel state (below the phase transition temperature), the liposome membrane is relatively rigid and impermeable because lipid molecules are ordered and condensed with fully extended hydrocarbon chains. However, upon heating to above the lipid’s T_c_, the membrane becomes more fluid and permeable as the mobility of the lipid head groups gradually increases, allowing the encapsulated drugs to escape. The initial thermosensitive liposomes were primarily based on DPPC (Tm = 41.4 °C) and DSPC (Tm = 2.5–44.5 °C) [60]. An alternative method to make liposomes responsive to temperature involves integrating synthetic polymers that disrupt the membrane when heated, such as poly(*N*-isopropylacrylamide) and poloxamers.

Sabbagh et al. [61] investigated 5-FU delivery via thermosensitive stealth liposomes designed to trigger drug release upon the application of mild hyperthermia using focused ultrasound. Liposomes composed of 1,2-dipalmitoyl-sn-glycero-3-phosphocholine (DPPC)/CHOL/DSPE-PEG at a 90:5:5 mol % were selected as a thermosensitive platform because DPPC exhibits a phase transition temperature of 41.5 ± 0.5 °C. At a temperature of 42 °C, the release of 5-FU from the thermosensitive liposomes was 68% when using focused ultrasound, within a period of 10 min. When the thermosensitive stealth liposomes were assessed in terms of their cytotoxicity on HT-29 human colon cancer, the IC_50_ reduced from 115 down to 49 μM. Pharmacokinetic studies in HT-29 tumor-bearing mice confirmed that the liposomal formulation circulated longer than the free drug, indicating stability at body temperature.

Clares et al. [62] developed magnetoliposomes containing superparamagnetic magnetite (Fe_3_O_4_) particles embedded into a phosphatidylcholine (PC)-based liposome (3:4 mass ratio, respectively) and loaded with 5-FU. The magnetic particles could be heated by applying an alternating magnetic field, which induced hyperthermia at the target site. The liposomal formulation exhibited rapid release profiles under mild hyperthermia. The in vitro release of the entrapped 5-FU was about 45% after 1 h. It is suggested that, when exposed to hyperthermia, the interface between the solid and fluid areas within the liposome structure becomes disrupted. This disturbance leads to the enhanced movement of phospholipids, thus increasing the release of 5-FU. In addition, the in vitro cytotoxicity data of blank (drug-unloaded) magnetoliposomes on human colon carcinoma T-84 cell lines exhibited a negligible cytotoxicity, indicating that these magnetoliposomes can be considered safe for parenteral administration.

Zhou et al. [63] explored a multifunctional stimuli-responsive drug delivery system by preparing a thermosensitive liposome encapsulating bismuth nanosheets, metformin, and 5-FU. The bismuth nanosheets functioned as the photothermal agent that disrupted the structure of the temperature-sensitive liposomes, leading to drug release. The liposomes were prepared using a reverse-phase evaporation method. Amounts of 3.9 mg of aqueous metformin, 3.9 mg of aqueous 5-FU, and 500 μL of a bismuth nanosheet solution were added to liposomal lipids (dipalmitoylphosphatidylcholine (DPPC)/cholesterol/DSPE-PEG2000 in a ratio of 86:10:4) dissolved in chloroform. Under the effect of mild hyperthermia (42 °C), the release rate of 5-FU after 24 h was 89% compared to 20% when the temperature was below 40 °C. The in vitro cytotoxicity assays demonstrated that, when combined with NIR laser irradiation, thermosensitive liposomes encapsulating 5-FU resulted in a significantly lower survival rate of HT29 cell lines (22.7%) compared to single antitumor treatments. Furthermore, the in vivo antitumor studies showed that thermosensitive liposomes encapsulating 5-FU produced smaller tumor volumes compared to the free metformin and 5-FU.

Novel formulations of thermosensitive liposomes have been shown to be effective drug delivery systems for various chemotherapeutic agents, and they may exhibit promising methods for 5-FU delivery to CRC sites (Table 2). Incorporating lysolipids, such as 1-palmitoyl-2-hydroxy-sn-glycero-3-phosphocholine (MPPC) and 1-myristoyl-2-stearoyl-sn-glycero-3-phosphocholine (MSCP), into PEGylated DPPC liposomes resulted in a more rapid drug release compared to traditional DPPC-based thermosensitive liposomes. Needham et al. [64] investigated lysolipid-containing thermosensitive liposomes composed of DPPC/MPPC/DSPE-PEG-2000 in a molar ratio of 90:10:4. The lysolipid-containing liposomes released approximately 45% of the doxorubicin contents within 20 s when exposed to 42 °C, whereas the DPPC liposomes released only 20% over 1 h. An in vivo study using a human squamous cell carcinoma tumor xenograft model (FaDu) demonstrated that the doxorubicin levels extracted from tumors were significantly higher in animals treated with both MPPC-containing thermosensitive liposomes and mild hyperthermia. Lysolipids are prone to forming highly curved micelles, which maintain defects in the membrane bilayer as the phase transition temperature is neared. Lindner et al. [65] developed thermosensitive liposomes based on phosphatidyloligoglycerol (DPPGOG) to prolong the DOX half-life in vivo without using the PEG lipid. The liposomal formulation, composed of DPPC/DSPC/DPPGOG in a ratio of 50:20:30 (m/m), effectively encapsulated DOX and almost completely released its DOX contents within 2 min at 42 °C. 

Another method for making liposomes temperature-sensitive is to incorporate synthetic polymers that become membrane-disruptive when heated. Temperature-sensitive polymers are water-soluble and typically form a coil structure below their lower critical solution temperature (LCST) due to hydrogen bonding between polymer chains. This hydrogen bonding allows water molecules to solubilize and maintains the polymer in a hydrated coil state. However, as the temperature approaches the LCST, hydrogen bonding becomes insufficient for solubilization. Consequently, the polymers become water-insoluble, transitioning to a dehydrated (hydrophobic) globule state, which destabilizes liposomes and facilitates the release of their payloads [66].

**Figure 3:**
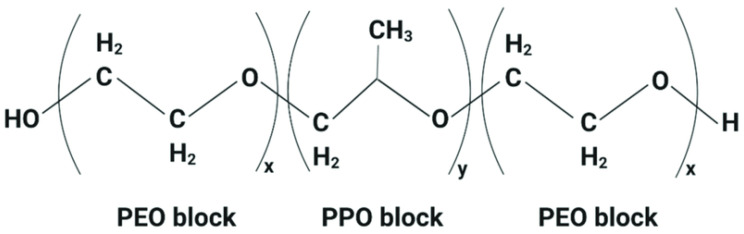
The general chemical structure of a poloxamer.

Poloxamers, also known as Pluronics, are a class of nonionic triblock copolymers composed of a central hydrophobic block of polypropylene oxide (PPO) flanked by two hydrophilic blocks of polyethylene oxide (PEO) (Figure 3). They exhibit temperature-dependent solubility and phase behavior, making them useful in thermosensitive drug delivery systems [67]. Oxaliplatin thermosensitive liposomes, consisting of DPPC, MSPC, poloxamer 188, and DSPE-PEG2000 (85:9.5:0.5:5, molar %), significantly accelerated the release of oxaliplatin when the temperature was around 42 °C, achieving a cumulative release of 90% within 10 min [68]. Thermosensitive oxaliplatin liposomes accelerated the release of oxaliplatin significantly when the triggered temperature was around 42 °C; the cumulative release of oxaliplatin reached 90% at 10 min. The antitumor activity of thermosensitive liposomes (2.5 mg/kg) was comparable to an oxaliplatin injection and non-thermosensitive liposomes at 5 mg/kg.

Thermosensitive poly(N-isopropyl acrylamide) (PNIPAM) liposomes are an advanced drug delivery system designed to release their encapsulated drugs in response to temperature changes. PNIPAM is a well-known thermosensitive polymer that exhibits a lower critical solution temperature (LCST) of around 32 °C. However, the lower critical solution temperature (LCST) of 32 °C makes this polymer impractical for clinical use. To achieve an LCST within the physiological temperature range, NIPAAm can be copolymerized with monomers such as hydrophilic acrylamide (AAm). The copolymerization of NIPAAm with different molar concentrations of AAm proportionally raises the LCST of NIPAAm based on the AAm concentration [69]. For instance, copolymers of 10% AAm increase the lower critical solution temperature from 32 °C to 39 °C, whereas 20% AAm demonstrated a lower critical solution temperature of 47.2 °C. Han et al. [70] investigated the release profile of doxorubicin using NIPAAm-AAM (83:17 mol with an LCST of 40 °C). A liposomal formulation consisting of DPPC/HSPC/CHOL (100:50:30:6) conjugated with NIPAAm-AAM (83:17) at a final concentration of 10 mg/mL released approximately 62% of the encapsulated doxorubicin at 40 °C. When the surrounding temperature exceeded the LCST of PNIPAM, the polymer chains in the liposome membrane became hydrophobic, causing the membrane to destabilize. Poly(*N*-vinylethers) polymers have been studied for the temperature sensitization of liposomes. They obtain their thermosensitive features from the dehydration of polymer chains, exhibiting a similar mechanism to that of NIPAAm-based copolymers. Kono et al. [71] fabricated poly[2-(2-ethoxy) ethoxyethyl vinyl ether] (EOEOVE), which had a lower critical solution temperature of around 40 °C, and used PEGyated liposomes encapsulated with doxorubicin to improve the antitumor activity against mouse colon carcinoma. The liposomal formulation composed of EYPC/Chol/PEG-PE (50:45:4) and modified with 2 mol% EOEOVE significantly increased the doxorubicin release above 40 °C, achieving complete release within 1 min at 45 °C. In vivo studies have shown that doxorubicin remained encapsulated within the liposomes. However, when mild heat was applied, the drug was effectively released, resulting in suppressed tumor growth in mice compared to the same liposomal formulation without the polymer. In PEGylated thermosensitive liposomes, DSPE-mPEG2000 can be substituted with nonionic surfactants containing PEGylated acyl chains, such as stearyl ether (Brij78), to improve the pharmacokinetics [72]. A DPPC lipid and Brij78 (96:4, molar ratio) liposomal formulation loaded with doxorubicin was reported by Tagami et al. [73]. The doxorubicin release from the formulation was significantly faster at 40–41 °C (100% release in 2–3 min).

**Table 2:** Some lipids and polymers used to prepare thermosensitive liposomes.

Material	Formulation Composition	Drug	Phase Transition Temperature (Tm)	Refs.
DPPC	DPPC/DSPC, 9:1 + 3 mol% PEG	Doxorubicin	42 °C	[60]
MPPC	DPPC/MPPC/DSPE-PEG-2000 (90:10:4)	Doxorubicin	39–40 °C	[64]
DPPGOG	DPPC/DSPC/DPPGOG 50:20:30 (m/m)	Doxorubicin	42 °C	[65]
Poloxamer 188	DPPC, MSPC, poloxamer 188, and DSPE-PEG2000 (85:9.5:0.5:5, molar %)	Oxaliplatin	42 °C	[68]
PNIPAM	DPPC/HSPC/CHOL/DSPE-PEG-2000 (100:50:30:6) with NIPAAm-AAM (83:17) 10 mg/mL	Doxorubicin	40 °C	[70]
EOEOVE	EYPC/Chol/PEG-PE (50:45:4) + EOEOVE 2 mol%	Doxorubicin	45 °C	[71]
Brij78	DPPC/Brij78 (96:4 mol/mol)	Doxorubicin	41.0 °C	[73]

### 6.2. Magnetic Liposomes

Magnetic liposome drug delivery systems can be achieved if the nanocarrier possesses a strong magnetic feature and can be employed through the application of an external magnetic field. Magnetic liposomes offer the primary benefits of both targeting strategies: they do not require surface modifications such as passive targeting, yet they can be actively guided to a specific location, as in active targeting. Furthermore, magnetic materials used as a part of liposomes can serve as hyperthermia-inducing agents. The exothermic properties arise from hysteresis and Néel relaxation losses through the application of a high-frequency magnetic field, increasing the local temperature surrounding the cells [71]. The interaction between the magnetic field and magnetic nanoparticles (MNPs) is thought to create heat, which can raise the temperature of the lipid bilayer past its phase transition temperature, thereby triggering the release of drugs [74]. Lately, there has been considerable interest in magnetic nanoparticles made from iron oxide (Fe_3_O_4_) for their use in magnetically triggered release systems in the biomedical field, a choice influenced by their physical characteristics and compatibility with biological systems [75]. Azevedo et al. [76] developed liposomes co-loaded with 5-FU and iron oxide (II, III) nanoparticles to maximize the preferential targeting and release of 5-FU toward colon cancer tumor sites. The liposomal nanosystem, composed of dimyristoyl phosphatidyl choline (DMPC)/Chol/DSPE-PEG (85:10:5), was able to respond to an external magnetic field (560 mT), as the in vitro concentration of 5-FU increased near the magnet site over the time (0–8 min) when compared to data far from the magnet site. In vitro cytotoxicity experiments on the CT-26 colon cancer cell line showed that the cytotoxic effect of 5-FU remained the same whether it was solely encapsulated in liposomes or jointly loaded with iron oxide (II, III) nanoparticles, indicating that iron oxide did not induce any additional cytotoxicity.

Doxorubicin-loaded magnetic liposomes, composed of DPPC/Chol/DHSG/DSPE-PEG5000 (5/4.85/1.04/0.03 molar ratio) and hydrated 2 mg/mL dextran-coated magnetic nanoparticles, exhibited selective liposome cell internalization with a hepatocellular carcinoma cell line (HepG2) corresponding to the magnetic field [77]. Furthermore, the encapsulation of dextran-coated magnetic nanoparticles inside liposomes enables MRI tracking of the formulation during its biodistribution. Magnetic liposomes are frequently conjugated with targeting moieties to achieve ligand-mediated active targeting. The ligand guides the liposomal assembly to the target site and promotes internalization into cancer cells, resulting in higher intracellular concentrations. For instance, the binding of hyaluronic acid to CD44 receptors on the tumor’s surface enabled the rapid internalization of the formulation. Magnetic liposomes loaded with doxorubicin and coated with hyaluronic acid showed a higher accumulation and targeting of doxorubicin in human glioblastoma cells (U87) [78]. Magnetic liposomes are frequently combined with another stimulus to enhance the likelihood of stimuli-sensitive release at the targeted tumor site. Anilkumar et al. [79] developed magnetic photosensitive liposomes by coating them with hyaluronic acid and encapsulated indocyanine green (ICG) as a photosensitizer molecule to facilitate photoactivation. After treating human glioblastoma cells (U-87MG) with the dual-targeted liposomes, the in vitro cell culture experiments confirmed an enhanced cytotoxicity after 4 min of exposure to a near-infrared laser. Pradhan et al. [80] designed folate-receptor-targeted thermosensitive magnetic liposomes (DPPC/cholesterol/DSPE-PEG(2000)/DSPE-PEG(2000)–folate in an 80:20:4.5:0.5 molar ratio) to enhance the doxorubicin cytotoxicity against the KB and HeLa cell lines. The liposomal formulation yielded a considerable increase in the cellular uptake of doxorubicin when compared to commercially available liposomal doxorubicin (Caelyx), free doxorubicin, and non-magnetic folate-targeted liposomes.

### 6.3. Ultrasound-Responsive Liposomes

Ultrasound-responsive liposomes are a sophisticated type of drug carrier that release their contents when stimulated by ultrasound. This responsiveness to ultrasound allows for a targeted approach to therapy, which is particularly useful in treating localized diseases such as cancerous tumors [81]. By utilizing ultrasound waves, responsive liposomes can be prompted to release their drug payload through a variety of mechanisms. (i) The mechanical stimulation from the waves may disrupt the liposome membrane, causing the drug to be released [82]; (ii) at the same time, thermal activation from the ultrasound’s heat can make the liposome membrane more permeable, leading to drug leakage [83]; and (iii) the process of acoustic cavitation, where the ultrasound leads to the formation and implosion of minuscule bubbles, produces shock waves that can trigger the liposomes to release their cargo [84]. Ezekiel et al. [85] reported crude soy echogenic lecithin liposomes encapsulating 5-FU, which were produced using the thin-film hydration method, as a potential liposomal formulation to treat colorectal cancer. The release pattern of 5-FU was evaluated under the influence of 20 kHz low-frequency ultrasound waves at various amplitudes and exposure times. Upon investigating various amplitudes of 10%, 15%, and 20%, it was observed that the release of 5-FU increased proportionally with the rise in amplitude. Notably, around 65% of the 5-FU was released after 10 min at the maximum amplitude applied. Without ultrasound application, the total amount of 5-FU released from the liposomal formulation after 12 h was approximately 70%.

In another study, ultrasound-responsive liposomes, composed of DSPC/DSPE-PEG/cholesterol/DOPE/MSPC, were fabricated and used as drug carriers for delivering doxorubicin (DOX) [84]. Under ultrasound irradiation with a 92 W/cm^2^ intensity at 24 kHz, the liposomal formulation showed a DOX release of 58.8%. The combination of liposomes and focused ultrasound demonstrated significant therapeutic effectiveness in MDA-MB-231 tumor-bearing mice. DOX released from the liposomes accumulated in the tumor tissues, killing the cancer cells and resulting in tumor suppression. Ultrasound-responsive liposomes could facilitate the delivery of various therapeutic agents into specific areas of the body [86,87]. Since a wide variety of therapeutic agents can be encapsulated into liposomes, ultrasound-responsive liposomes can serve as a versatile platform for numerous medical applications.

### 6.4. Photo-Triggerable Liposomes

**Figure 4:**
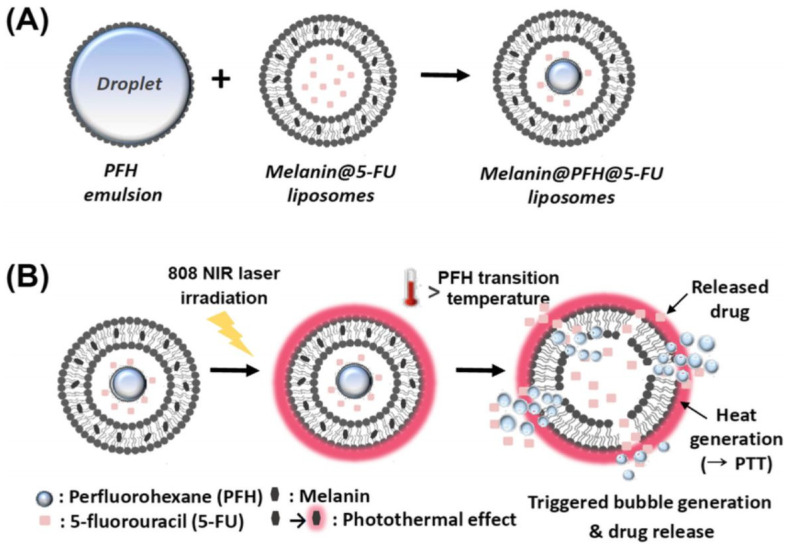
(**A**) Schematic illustration of preparation of melanin-PFH-5-FU photo-triggerable liposomes; (**B**) photothermal effect of melanin accelerates 5-FU release through vaporization of PFH [93]. (Reprinted with permission from Springer Nature. Higher resolution/color version of this figure is available in electronic copy of article).

Liposomes that are loaded with photoactive molecules, specifically known as photosensitizers, are gaining tremendous attention for their use in the design of photo-triggerable drug delivery systems [88]. Photo-triggerable liposomes are specialized drug delivery vehicles that release their contents when exposed to light, typically of a specific wavelength [89]. Near-infrared (NIR) light presents a compelling method for externally triggered drug release because of its deeper tissue penetration and inherent harmlessness, offering precise control over both the location and timing of the drug release [90]. When the target site is exposed to light, usually in the NIR range for better tissue penetration, the photosensitizers absorb the light energy. The absorbed energy causes the photosensitizers to enter an excited state. In this state, the energy can be transferred to surrounding oxygen molecules, producing reactive oxygen species (ROS) [91]. The generated ROS can destabilize the liposome membrane, leading to a change in its permeability and the release of the therapeutic agent contained within [92]. Kim and Lee [93] developed photothermal liposomes containing melanin, as a photoactive agent, and perfluorohexane for the controlled release of 5-FU. The drug release behavior of liposomes due to the photothermal effect of melanin was evaluated with an 808 nm NIR laser at a power density of 1.5 W/cm2 for 10 min (Figure 4). The liposomes released 54.1% of the loaded 5-FU at 24 h compared to 31.8% without laser irradiation. The in vitro cytotoxicity of the liposomes was evaluated using CT26 murine colorectal cancer cells (CT26). The liposomes had no cytotoxicity without laser irradiation; however, the liposomes were toxic to CT26 cells upon exposure to laser irradiation. The tumor growth in mice injected with liposomes under laser irradiation was inhibited significantly compared to mice injected with liposomes that were not exposed to laser irradiation. 

The utilization of light as a stimulus is a commonly investigated non-invasive approach to initiate the release of liposome-encapsulated chemotherapeutic agents. Light-sensitive liposomes can be prepared using photo-polymerizable phospholipids such as DC8,9PC (1,2-bis(tricosa-10,12-diynoyl)-sn-glycero-3-phosphocholine). Photo-triggerable liposomes containing DPPC/DC(8),(9)PC/DSPE-PEG2000 (86:10:04 molar ratio) were developed as delivery vehicles of doxorubicin [94]. A laser treatment (5 min at 514 nm) on liposomal doxorubicin resulted in at least a 2–3-fold increase in cell growth inhibition compared to untreated cells after 48 h against the Raji and MCF-7 cell lines. Guo et al. [95] incorporated UV cross-linkable 10,12-pentacosadiynoic acid (PCDA) into phospholipid bilayer vesicles as adjustable additives for photo-release applications. The polymerization of PCDA monomers occurred between adjacent diacetylene groups in the membrane to release encapsulated molecules upon UV irradiation. The paclitaxel release reached 90.5 ± 3.7% within 24 h following 20 min of irradiation. It is worth noting that increasing the PCDA molar ratio and the irradiation time will decrease the cumulative drug release because an increased degree of polymerization will block the channels of drug-loaded polymerized vesicles. The chlorin-based photosensitizer chlorin e6 (Ce6) incorporated into liposome membranes can disrupt the phospholipid bilayers upon light exposure, enabling the controlled release of the encapsulated contents. Doxorubicin (DOX) and Ce6 were co-encapsulated in liposomes to explore the synergistic cytotoxicity effects [96]. The DOX-Ce6 liposome showed more than a 3-fold increase in cytotoxicity upon irradiation against different cell lines (A375 and C26), and the in vivo results indicated a lower tumor growth rate compared to liposomal DOX and liposomal Ce6 alone. 

NIR-absorptive stealth liposomes containing DOX and the heptamethine indocyanine dye IR825 were used for a combined photothermal and chemotherapeutic cancer treatment [97]. This combined approach significantly enhanced the HeLa cell killing efficiency, resulting in less than 20% cell viability after 5–10 min of NIR irradiation (808 nm, 0.5 W/cm²). Porphyrins are employed in clinics as photosensitizers for cancer treatment. Porphyrin-based photosensitizers have been incorporated into liposome formulations and studied for their potential photocytotoxic effects. Feng et al. [98] developed hyaluronic acid-grafted liposomes containing porphyrin as a photosensitizer against MDA-MB-231 cells and observed that these liposomes exhibited the highest toxicity to the cells upon light exposure. Light-mediated release methods, which might be promising for efficient 5-FU delivery, include a photoresponsive mechanism with phthalocyanine, 2-(1-hexyloxyethyl)-2-devinyl pyropheophorbide-a (HPPH) [99], 7-acetoxycoumarin dimer (ACD) [100], merocyanine 540 [101], photochemical nitric oxide (NO) precursors [102], azobenzene motifs as light-responsive membrane-interactive compounds [103], and m-THPC (meta-tetra[hydroxyphenyl]chlorin) [104].

## 7. Conclusions and Future Perspectives

The introduction of innovative drug delivery systems (DDSs) in pharmaceutical sciences has transformed healthcare systems significantly. This is due to advanced carrier-based DDSs that are capable of significantly reducing the adverse side effects associated with medications while simultaneously enhancing their effectiveness. Liposomal drug products have proven to be effective nanocarriers in clinical settings, and several are currently popular in the pharmaceutical market due to their non-toxicity, biocompatibility, and surface-conjugation capabilities. To further enhance the safety and efficacy of liposomes, various structural modifications have been applied to them. Stimuli-responsive liposomes are an advanced form of liposomes that are designed to respond to specific triggers or stimuli in their environment. Temperature, magnetic energy, and light can be externally applied to liposome formulations, and such formulations have shown significant potential for clinical use. Internal stimuli (e.g., pH and enzymes) provide numerous opportunities in the development of liposomes to ensure maximum drug delivery efficiency in tumor sites without affecting the surrounding tissues. Stimuli-responsive liposomes have demonstrated a high potential in the release of chemotherapeutic drugs in tumor sites to considerably enhance the efficacy of these drugs in cancer suppression. 

5-FU stimuli-responsive liposomes have demonstrated their clinical use in CRC (Table 3). However, the efficacy of 5-FU stimuli-responsive liposomes at CRC sites can be further enhanced by designing multi-sensitive liposomes. Dual-targeting stimuli-triggered liposomes (e.g., light/pH, redox/pH) have several advantages, such as releasing the encapsulated drugs with a higher efficiency, a higher accuracy, and a better tumor cellular internalization. Developing innovative stimuli-responsive liposomes for 5-FU delivery remains an ongoing task to improve both the efficacy and safety of the drug. There are several strategies for the preparation of 5-FU stimuli-responsive liposomes that might have the potential to improve 5-FU delivery, reduce its systemic toxicity, and enhance its therapeutic outcomes. The surface modification of liposomes with biocompatible polymers that are destabilized at a weakly acidic pH and under mild hyperthermia may create novel therapeutic strategies for the site-specific and controlled release of 5-FU in response to external or internal stimuli. Redox-sensitive liposomes can be considered for developing 5-FU stimuli-responsive liposomes. The rationale behind creating redox-sensitive liposomes lies in attaching 5-FU to liposomes through disulfide linkages. When encountering elevated levels of glutathione at the tumor site, these bonds break, initiating the release of the medication [105,106]. Hypoxia-responsive liposomes are a specialized type of stimuli-responsive liposome designed to release their therapeutic contents in response to low-oxygen conditions, which are common in CRC sites. This can be achieved through several mechanisms, such as the incorporation of hypoxia-sensitive chemical moieties (e.g., nitroimidazole derivatives) that undergo reductive cleavage in low-oxygen environments [107].

Stimuli-responsive liposomes are highly safe and biocompatible; however, endogenous stimuli, such as enzyme or pH triggers, may exhibit considerable variability among different organs, patient age groups, and populations. A present challenge with liposomes activated by external stimuli lies in optimizing the delivery of drugs to various sites. Moreover, discrepancies in the data obtained from small to large animal models and humans present obstacles in the clinical application. Further research is required to understand how the tumor microenvironment is regulated and impacts the activity of stimuli-responsive liposomes.

## Figures and Tables

**Table 1:** Some lipids, polymers, and moieties utilized to form pH-sensitive liposomes.

Lipid/Polymer/Moiety Introduced for pH Sensitivity	Liposomal Composition	Drug	Refs.
Folate–PEG3350-CHEMS	HSPC/CHOL/mPEG2000-DSPE/folate–PEG3350-CHEMS (55:40:4:1)	Imatinib	[39]
OA	PE/CHOL/OA (3:2:3 *w*/*w*)	Docetaxel	[40]
Hydrazone	mPEG2000-Hz-CHEMS (90:10:3:3)	Paclitaxel	[41]
PDPA	HSPC or DOPC, Chol, and PEGm-PDPAn-PEGm in various molar ratios	Doxorubicin	[42]
PAA	DPPC/DOPG/cholesterol (18.048:1.152:12.8 μmol) + 10 mol% of Chol-PAA	Doxorubicin	[43]
Vinyl ether	DOPE:mPEG-VE-DOG (90:10 mol%)	Calcein	[44]
PIVE	PEG-PIVE/DOPE (2:98, 5:95, and 12:88 mPEG-PIVE/DOPE)	Calcein	[45]

**Table 3:** Lipids and materials used to prepare 5-FU stimuli-responsive liposomes.

Material	Liposomal Composition	Refs.
pH-Responsive Liposomes		
CHEMS	CHEM/CH/tween 20/DSPE-PEG2000 (60:20:10:10 molar ratio)	[33]
SMA	DSPC/SMA (20:1 molar ratio)	[34]
DOPE	DOTAP/DOPE/des PEG/HA (1:1:1:0.5 molar ratio)	[35]
Chitosan	HSPC/CH/DPPG (7:2:1 molar ratio) + CS, miR and siRNA, CS, and HA layers onto the surface	[37]
Enzyme-Responsive Liposomes		
sPLA_2_ enzyme	OFZG/CH/tween 80 (2:1:0.1 molar ratio)	[52]
Thermosensitive Liposomes		
DPPC	DPPC/CH/DSPE-PEG (90:5:5 mol%)	[61]
Fe_3_O_4_	Fe_3_O_4_/PC (3:4 mass ratio)	[62]
Bismuth nanosheets/DPPC	DPPC/CH/DSPE-PEG2000 (86:10:4 molar ratio) + BiNSs	[63]
Magnetic Liposomes		
Iron oxide I, II	DMPC/CH/DSPE-PEG (85:10:5)	[76]
Ultrasound-Responsive Liposomes		
Crude soybean lecithin	Crude soybean lecithin (75 mg) and cholesterol (25 mg)	[85]
Photo-Triggerable Liposomes		
Melanin	PC (10 mg), Chol (1.5 mg), DSPE-PEG (3 mg), and melanin (1 mg) mixed with PFH emulsion in a 1:1 ratio	[93]

## Abbreviations

AAm	hydrophilic acrylamide
CH	cholesterol
CHEMS	cholesterylhemisuccinate
CRC	colorectal cancer
CS	cationic chitosan
DC8,9PC	1,2-bis(tricosa-10,12-diynoyl)-sn-glycero-3-phosphocholine
DDSs	drug delivery systems
DHSG	1,5-*O*-dihexadecyl-*N*-succinyl-l-glutamate1,5-*O*-dihexadecyl-*N*-succinyll-glutamate
DMPC	dimyristoyl phosphatidyl choline
DOG	1,2-dioleylglycerol
DOPC	1,2-dioleoyl-snglycero-3-phosphocholine
DOPE	1,2-dioleoyl-sn-glycerol-3 phosphoethanolamine
DOTAP	1,2-dioleoyl-3-trimethylammonium-propane
DOX	doxorubicin
DPD	dihydropyrimidine dehydrogenase
DPPC	dipalmitoylphosphatidylcholine
DPPG	1,2-dipalmitoyl-sn-glycerol-3-phosphoglycerol
DPPGOG	phosphatidyloligoglycerol
DSPC	1,2-distearoyl-sn-glycero-3-phosphocholine
DSPE-PEG2000	1,2-distearoyl-sn-glycero-3-phosphoethanolamine-N-[amino(polyethyleneglycol)-2000] (DSPE-PEG2000)
EOEOVE	poly[2-(2-ethoxy) ethoxyethyl vinyl ether]
EYPC	egg yolk phosphatidylcholine
5-FU	5-fluorouracil
HA	sodium hyaluronate
HSPC	hydrogenated soybean phosphatidylcholine
HT29	human colorectal adenocarcinoma cells
MMPs	matrix metalloproteinases
MPPC	1-palmitoyl-2-hydroxy-sn-glycero-3 phosphocholine
MSPC	1-myristoyl-2-stearoyl-sn-glycero-3-phosphocholine
NHE1	Na^+^/H^+^ exchanger isoform 1
NIR	near-infrared
OA	oleic acid
OFZG	1-*O*-octadecyl-2-(5-fluorouracil)-*N*-acetyl-3-zidovudine-phosphorylglycerol
PAA	poly(acrylic acid)
PC	phosphatidylcholine
PCDA	10,12-pentacosadiynoic acid
PDPA	poly(2-(diisopropylamino) ethylmethacrylate)
PE	phosphatidylethanolamine
PFH	perfluorohexane
PIVE	phenyl-substituted vinyl ether
sPLA_2_	secretory phospholipase A_2_
PNIPAM	poly(N-isopropyl acrylamide)
PTX	paclitaxel
ROS	reactive oxygen species
SMA	poly(styrene-co-maleic acid)
S100PC	soybean phosphatidylcholine
T_c_	transition temperature
TME	tumor microenvironment
TS	thymidylate synthase
